# Personalized p-THYROSIM model for thyroid hormone dynamics, hypothyroidism treatment & implementation in an iOS version for wide distribution

**DOI:** 10.3389/fendo.2025.1735282

**Published:** 2026-01-09

**Authors:** Joseph DiStefano, Katarina Reid, Karim Ghabra, Rita Chen, Shruthi Sathya Narayanan

**Affiliations:** Biocybernetics Laboratory, Departments of Computer Science and Medicine, University of California at Los Angeles (UCLA), Los Angeles, United States

**Keywords:** math model, simulation model, residual thyroid function, optimal dosing, personalized medicine, monotherapy, combination hormone replacement therapy

## Abstract

**Objectives:**

To: (1) update and refine the predictive abilities of original p-THYROSIM, a uniquely personalized simulation tool that mathematically mimics the thyroid hormone regulation system in humans, to optimize replacement LT4 and LT4+LT3 dosing for hypothyroid patients, based on individual hormone levels, heights (*H*), weights (*W*) and sex. Refinements include dependence on *H* and *W* separately, rather than composite *BMI=W/H^2^*. And to: (2) present a new and user-friendly software tool, an iOS p-THYROSIM app for the iPhone and iPad, to accomplish these goals, as well as a Python version for longer term disease progression simulations.

**Methods:**

Original p-THYROSIM was refined and updated, first by refitting male and female data for establishing blood volume *V_b_* as a function of *W*s and *H*s of males and females separately. A superfluous parameter was also removed and FT4 and FT3 output plotting were slightly adjusted to align them with current assay ranges. We also developed two software packages for simulating the model: (1) a 100-day iOS implementation for the iPhone and iPad using Apple developer tools; and (2) a 1000-day version in Python, with time units converted from hours to days to render it more practical for research use with clinical diseases that evolve over months and years.

**Results:**

The iOS app was implemented, exercised and tested in several clinical applications. Most notably, simulation results are shown and compared for hemi-thyroidectomy and for optimal dosing for mono- and combination hormone replacement therapies. With the Python version, graphic hormone responses for 240 days of evolving mono- and combination replacement therapies were illustrated for a simulated Hashimoto’s disease patient. With both versions, simulated combination therapy was shown to be more effective in achieving normal range FT4, FT3 and TSH concentrations in plasma.

**Conclusions:**

p-THYROSIM can predictively provide accurate mono- and combination LT4+LT3 replacement hormone therapies for male and female hypothyroid patients, personalized with their *H*s and *W*s. Where combination therapy is warranted, our results predict that not much LT3 (typically 5 – 7.5 ug) is needed in addition to LT4 to restore euthyroid levels, with larger LT3 doses rarely needed, suggesting opportunities for further research exploring safe and effective combination therapy with lower T3 doses and slow-releasing T3 formulations.

## Introduction

**Figure 1:**
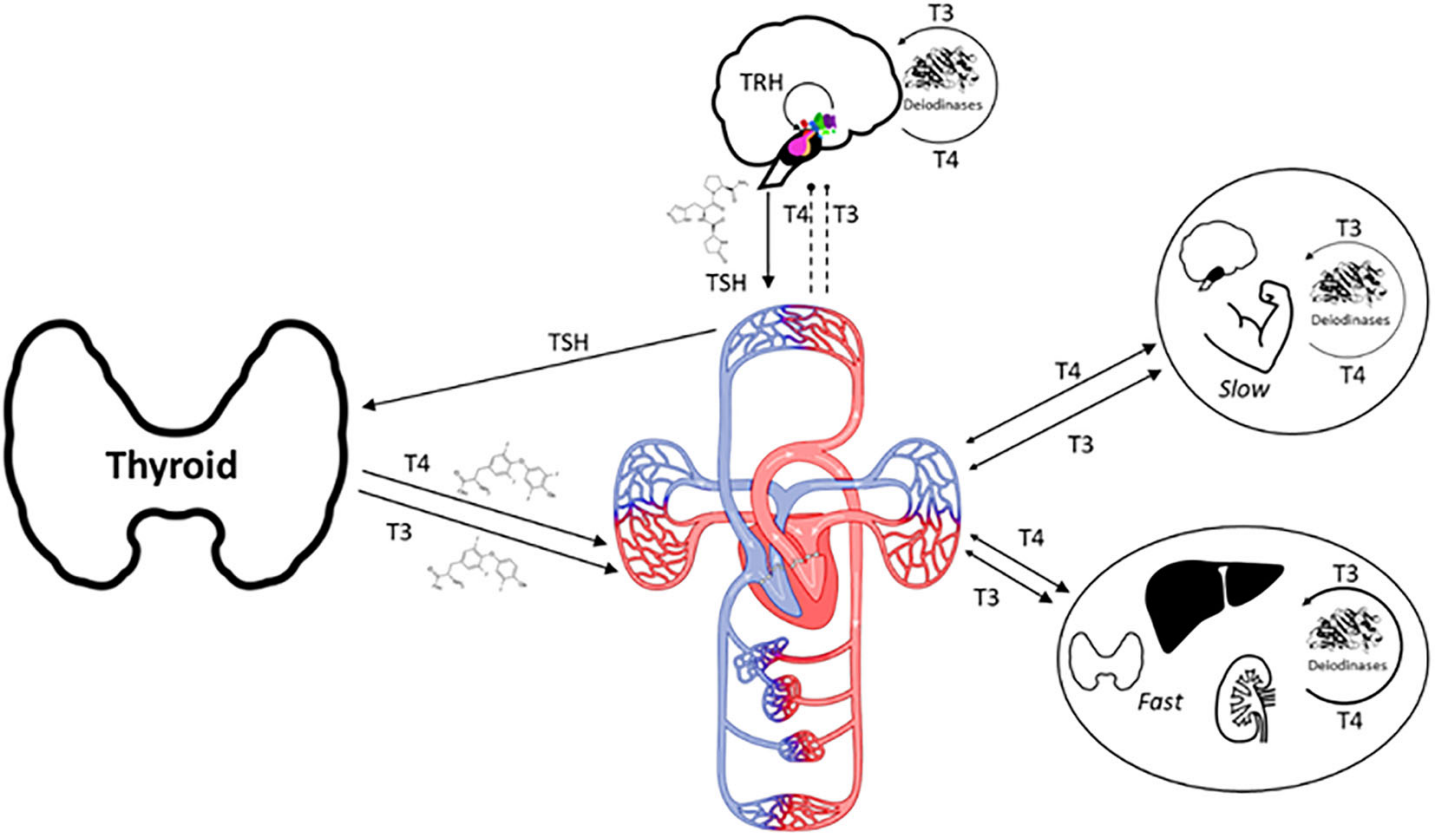
A physio-anatomic rendition of thyroid hormone (T4 and T3) productions, exchanges and regulation by pituitary hormone TSH and hypothalamic hormone TRH in humans. 100% of T4 and ~20% of T3 is produced and secreted by the thyroid gland, the remaining 80% of T3 is produced by enzymatic deiodination from T4 in every tissue, dynamically exchanging in rapidly (Fast tissues) and slowly (Slow tissues) with different tissue groups, as shown in the figure (3).

A personalized simulation tool p-THYROSIM that mathematically mimics the thyroid hormone regulation system in humans (**Figure 1**) was developed in (1) and applied to better optimize replacement LT4 and LT4+LT3 dosing for hypothyroid patients, based on individual hormone levels, BMIs and sex. Compared with 3 other dosing methods, the accuracy of p-THYROSIM optimized dosages for LT4 monotherapy was better overall (53 vs. 44, 43 and 38%) including for extreme BMI patients (63 vs.~51% low BMI, 48 vs.~36 & 22 for high BMI). Optimal dosing for combination LT4+LT3 therapy and unmeasured residual thyroid functions (RTFs) were predictively computed with p-THYROSIM for males and females in low, normal and high BMI ranges, yielding daily T3 doses of 5 to 7.5 μg T3 combined with 62.5–100 μg LT4 for women or 75–125 μg LT4 for men. The LT3 in these dosages were in line with that recommended by thyroid organizations in the United States and Europe (20:1 to 13:1 ratios) (2).

In subsequent research and clinical application of p-THYROSIM we found that in simulations of extremely low and high BMI patients, the original model displayed a systematic misfit in simulated serum FT3 concentrations. Lean individuals tended to appear with higher-than-expected FT3 while obese individuals showed lower-than-expected FT3. This suggested a size/composition and sex-related mapping issue, which further analysis traced the problem to an error in fitting blood volume *V_b_* data in (1) – incorrectly fitted to composite male-female data, instead of separately to male and female data. We refitted this data, revised the model accordingly, and updated a few other model issues, all as described below. These changes also included our use of *H* and *W* independently in our new model, not as a composite BMI.

We fully implemented the updated model in a new iOS app for the iPhone and iPad, as described below, and applied it to optimal monotherapy and combination therapy replacement hormone dosing in our example results.

## Methods and results

### Refitting of blood volume data separately for males and females

Height *H* and weight *W*, primary anthropometric parameters depicting size differences among individuals, are routinely measured at patient visits. Blood volumes *V_b_* are complex functions of *H* and *W*, rising and falling with increases and decreases in body size. To incorporate these parameters into the original p-THYROSIM model (1), we developed a regression equation for blood volume*V_b_* from an ideal weight formula fitted to a published and composite dataset containing 80 men and 80 women of different body compositions, *H*s (in meters) and body weights (*BW*s (in *kg*), ranging from underweight to obese (4). *V_b_* was expressed as a function of % deviation *Δ_iBW_* from ideal weight relative to ideal weight, with *iBW*(*H, sex*) the ideal weight of a patient expressed as quadratic functions of *H* for each sex, all fitted to data in Figures 1 and 3 of reference (4)). Plasma volume *V_P_* then was computed as *V_P_*=*V_b_*(1 – *HEM*), with hematocrit *HEM* approximated as 0.4 for female patients and 0.45 for male patients.

**Figure 2:**
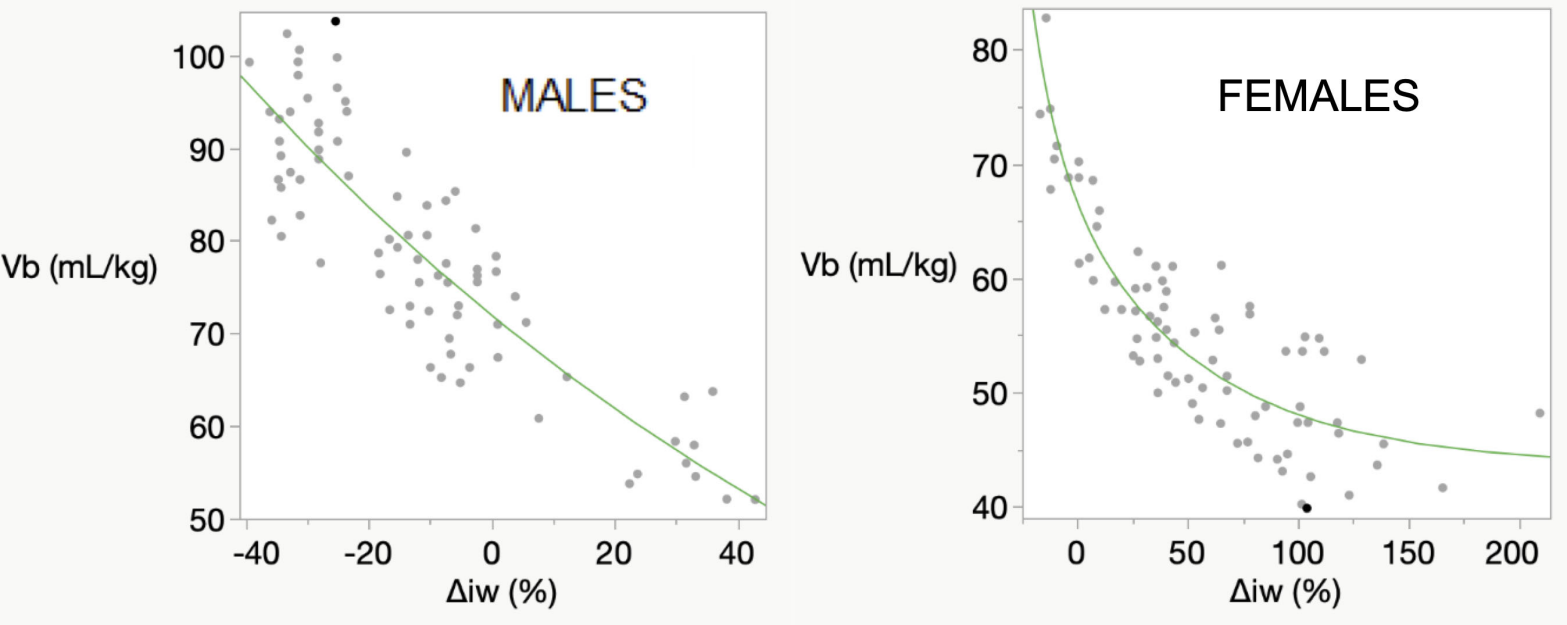
Male and female data from (4) refitted separately for males and for females. The abscissa deviation from ideal body weight Δiw (abbreviated from ΔiBW) incorporates sex-height *H* relationships as well as *W* for fitting *V_b_* to the published data.

**Figure 3:**
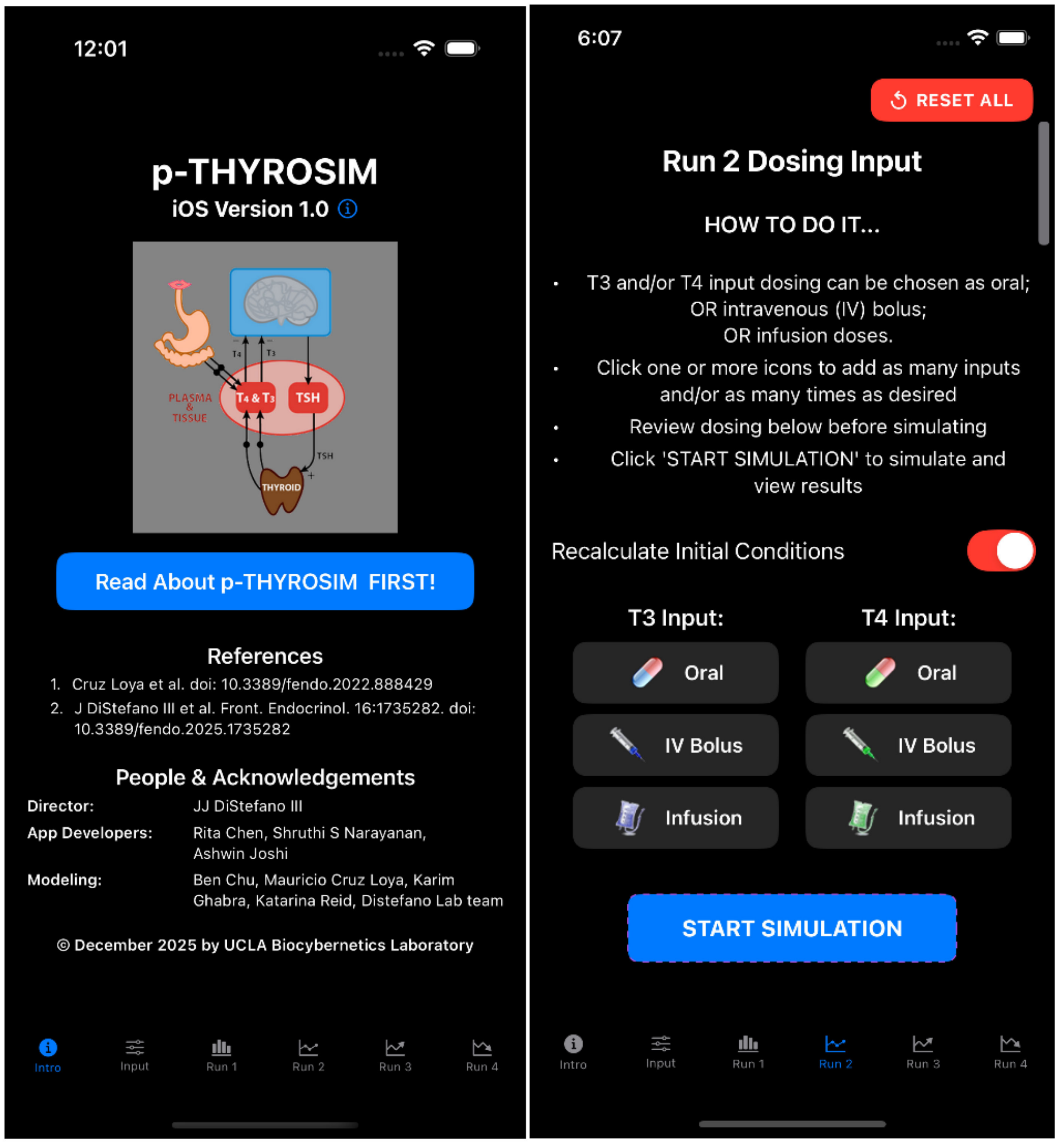
p-THYROSIM Introduction and Dosing Input choice pages.

Figure 3 in reference (1) was misfitted, as it did not capture the sex differences in blood volumes. These regressions were redone for female and male data separately, fitting *V_b_* for females from only female data (R^2^ = 0.79) and *V_b_* for males (R^2^ = 0.76) from only male data. The results are given in **Figure 2**.

MALE FITTED FUNCTION:


$$V_{b}=71.96e^{-0.007516\text{ * Δiw}}$$

FEMALE FITTED FUNCTION:


$$V_{b}=43.65+20.79e^{-0.01545\text{ * Δiw}}+2.043e^{-0.08392\text{ * Δiw}}$$

We also simplified the model by eliminating unnecessary allometric scaling in the formula for T3 degradation (*k*_05_) in the rapidly exchanging T3 compartment of the model but retained its small dependence on sex differences (about 5% higher in males). Together, these two model modifications eliminated the aberrational simulation response results at extreme BMI.

We also slightly adjusted the FT4 and FT3 output plotting functions to align them with current assay ranges in the UCLA Clinical Laboratory database, the primary source of our human data for modeling and applications of p-THYROSIM. Example results are illustrated below in the software iOS “app” development section below.

### iOS implementation of the model for the iPhone and iPad

Original (non-personalized) THYROSIM was developed as a web application (*biocyb1.cs.ucla.edu/thyrosim/cgi-bin/Thyrosim.cgi*) and also as an iOS version available from the Apple Store (search for: *Thyrosim*). Our first implementation of p-THYROSIM for distribution – described below, is an iOS version, with units in hours, for simulations up to 100 days (**Figure 3**). The code is here: tinyurl.com/pthyrosim. **Figure 3** also includes a picture of the dosing input page, with instructions and several input options.

The p-THYROSIM iOS app was built using the *Swift* language (5) and *SwiftUI* framework (6), creating a native mobile application that allows users (e.g. healthcare professionals) to simulate thyroid hormone treatments and predict patient responses to these treatments. The app serves as a clinical decision support tool, enabling users to model different dosing strategies (as in **Figure 3****RIGHT**) for patients with thyroid disorders before implementing actual treatments.

**Figure 4:**
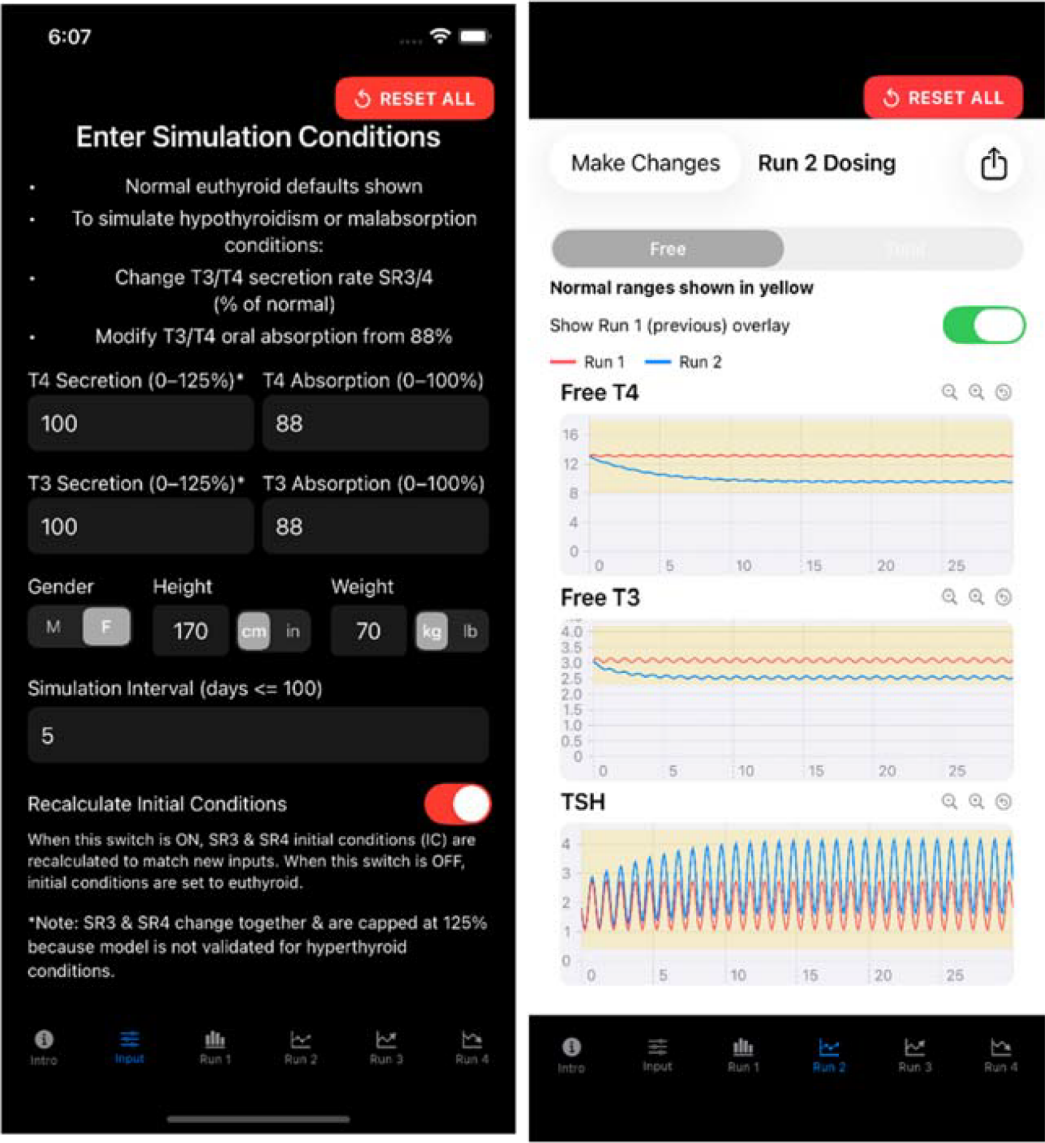
Input values (LEFT) and simulation (RIGHT) hormone response output pages of p-THYROSIM for a 70 kg 170 cm male under simulated hemi-thyroidectomy conditions (secretion rates 50%) with no replacement therapy, superimposed on prior euthyroid responses (secretion rates 100%) over 30 days.

Users input thyroid secretion rate and intestinal absorption rate changes from default values, as well as patient characteristics (height, weight and gender) on the (Input icon) page of the app (**Figure 4****LEFT**). Simulation results for euthyroid and hemi-thyroidectomy conditions for this male are shown in **Figure 4** on the RIGHT.

**Figure 5:**
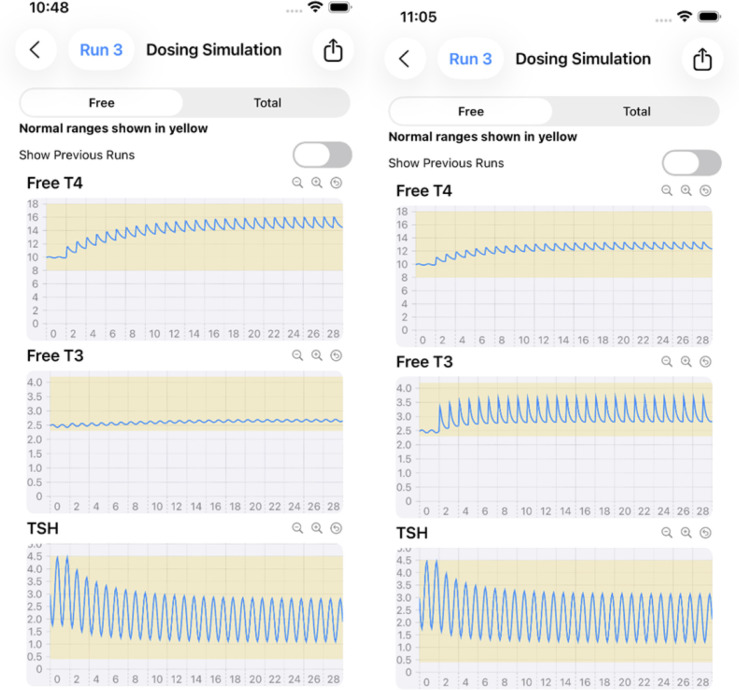
Monotherapy (75 μg LT4, LEFT) vs. combination therapy (50 ug LT4 + 7.5 μg LT3, RIGHT) in a 70 kg 170 cm male with 50% residual thyroid function (RTF=%secretion rates) over 30 days of treatment, with the goal of achieving mid-range hormone levels.

**Figure 6:**
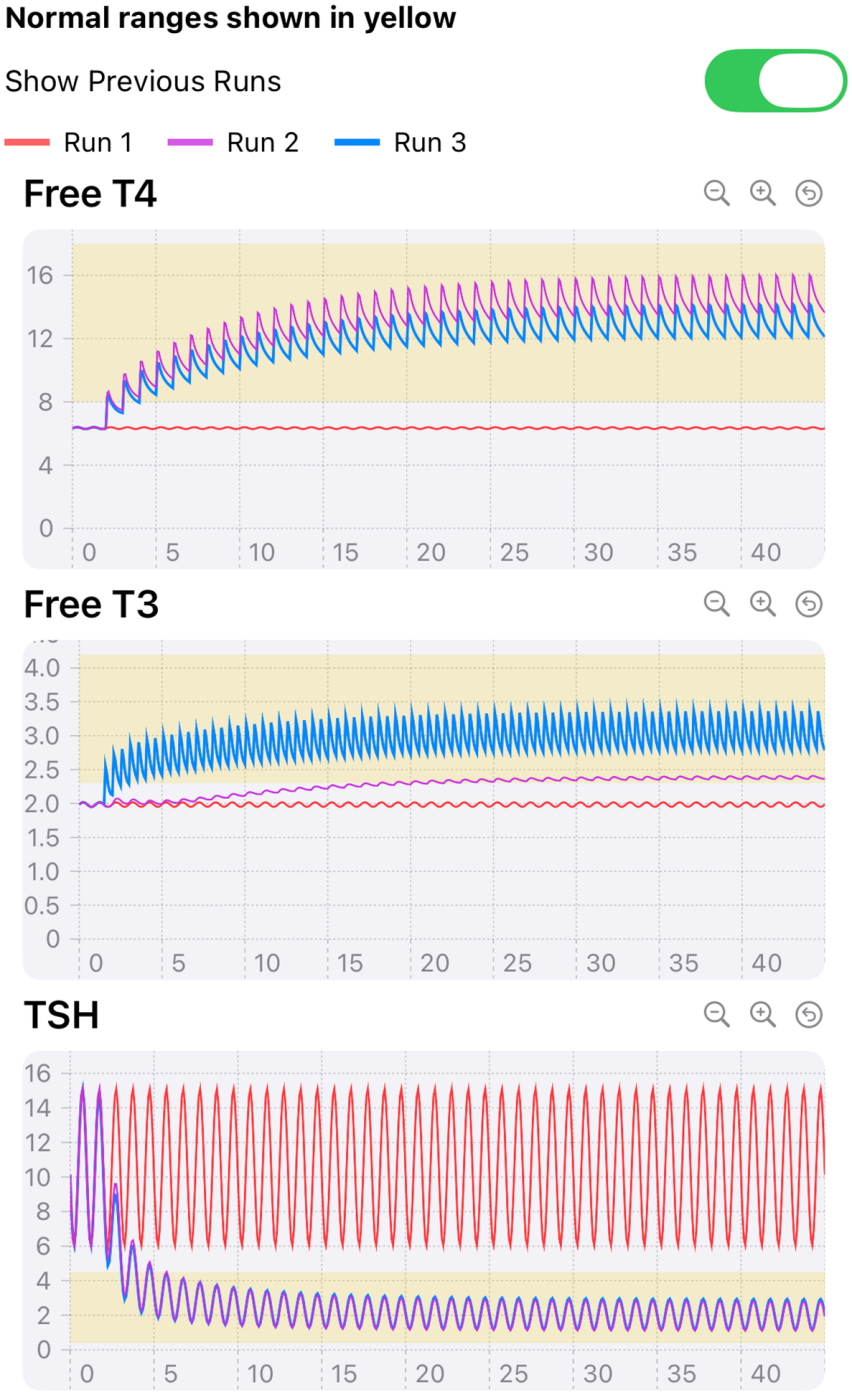
Illustration of 3 runs superimposed. Simulated 200 lb 65 inch female with 10% RTF receiving 0 (constant mean RED curves), 112.5 μg (TOP LT4 purple) LT4 only daily, and 100 μg LT4 once a day+5 ug LT3 *twice* daily (centered FT4 and high frequency FT3 peaks, BLUE) for 45 days. TSH curves with dosing are overlapped (BLUE and PURPLE), constant mean (RED) without dosing.

Various oral dosing scenarios (**Figure 3****RIGHT**) to visualize how hormone levels will change over time in response to these potential treatments are entered on the next few pages – the primary applications of the app, by clicking icons at the bottom of the page, as shown in **Figures 3** and **4**. Simulation results for other oral input choices are given graphically on subsequent pages (**Figures 5**, **6**). Up to four dosing strategies can be visualized simultaneously in a single graph (3 are shown in **Figure 6**).

The app translates a complex mathematical model into *Swift*, maintaining the same 19-compartment differential equation system that describes thyroid hormone dynamics (1). The core mathematical engine uses a 5th-order Runge-Kutta numerical solver that approximates the original Rodas5 solver from the scientific implementation. This solver integrates the complex physiological equations describing how FT4 (or TT4), FT3 (or TT3) and TSH hormones move through different body compartments, including plasma, various organs, under the control of pituitary-brain negative feedback mechanisms. Simulation output shows plasma hormone concentrations, the measurable compartments (as on the RIGHT in **Figure 4**).

The mathematical model runs behind the scenes, solving the differential equations with high precision to provide accurate predictions of hormone concentration changes over days or weeks. As one example in **Figure 5**, clinicians can optimize treatment dosing regimens for individual patients with the app. This figure provided a comparative example of *LT4 monotherapy versus LT4+LT3 combination therapy* in a single patient, each with the goal of achieving mid-range normal hormone levels. **Figure 5** results are shown in 2 graphs, for comparative clarity.

### Model and code adjustments for a longer-term (1000-DAY) Version of p-THYROSIM

Original p-THYROSIM was developed for simulations up to 100 days. To render it more practical for use with clinical diseases that evolve over months and years, we converted time units in the model from hours to days. To accomplish this, all fractional rate constants with the units per hour were converted to units of per day, by dividing them by 24. All parameters and variables with units of time in hours were converted to time in days by multiplying them by 24. This resulted in model simulations running about 24 times faster, and likely with less numerical errors. Python code (7, 8) for the 1000-day version is available at our Github website: tinyurl.com/pthyrosim.

**Figure 7:**
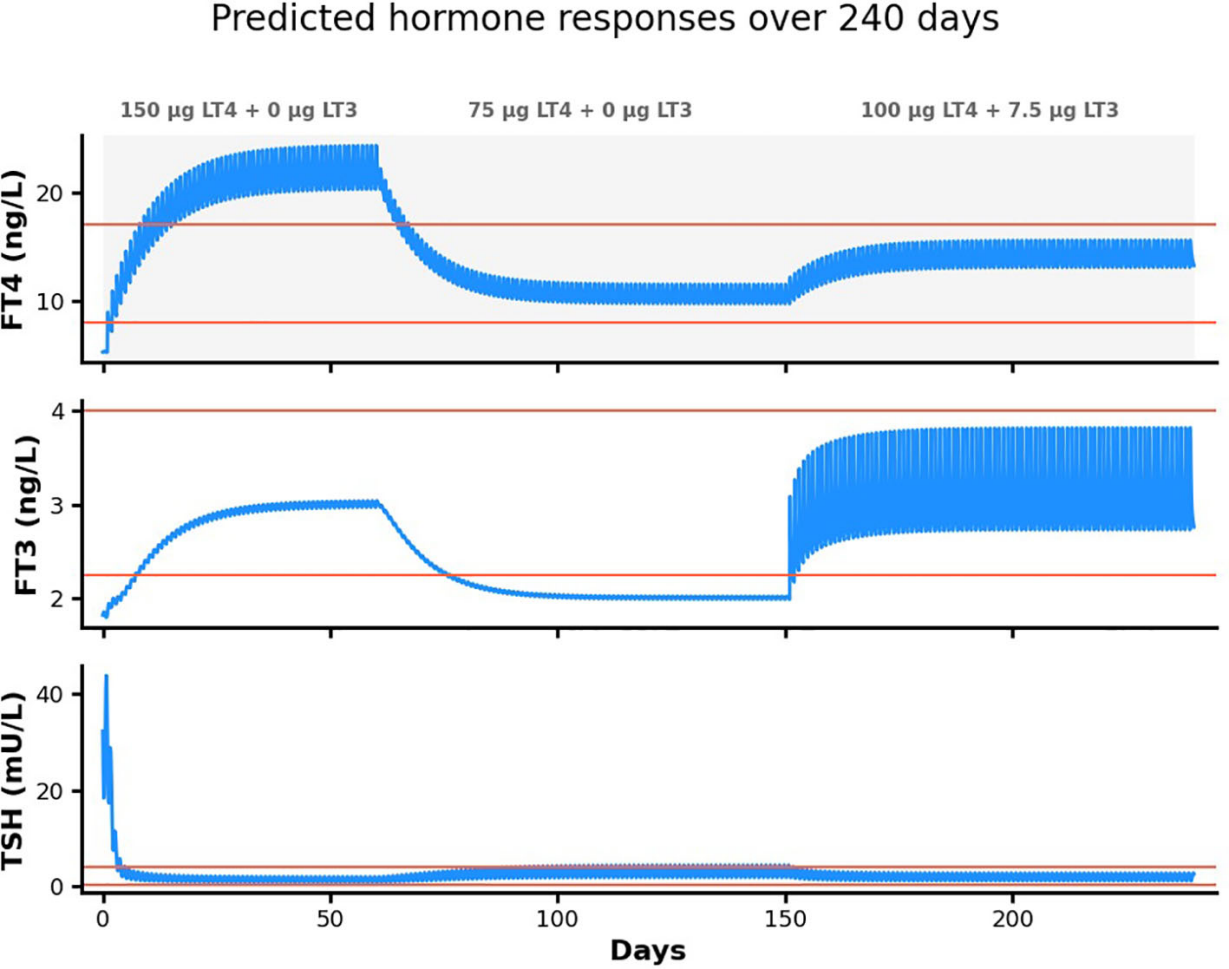
Simulation of 240 days of treatment with both monotherapy and combination replacement therapy in a simulated 54 kg 1.7m female Hashimoto’s patient. This hypothetical patient initially has TSH > 40 mU/L, FT4 = 5 ng/L and FT3 < 2 ng/L. She is started on 150 μg LT4 daily for 60 days, reduced to 75 μg LT4 daily for 90 days and finally given 100 μg LT4 + 7.5 μg LT3 daily for 90 days.

These temporal modifications allow for more realistic simulation of chronic thyroid conditions and their management, accommodating disease progression and therapeutic interventions over timeframes relevant to clinical practice. Furthermore, our revised parameterization ensures consistency with long-term clinical follow-up data, enhancing both the utility and reliability of p-THYROSIM for extended predictive modeling. Example plots from the 1000 day code are given in **Figure 7** for a simulated Hashimoto’s disease patient.

The simulated 54 kg 1.7m female patient initially has TSH > 40 mU/L, FT4 = 5 ng/L and FT3 < 2 ng/L. She is started on 150 μg LT4 daily for ~ 65 days, reduced to 75 μg LT4 daily for ~ 85 days and finally given 100 μg LT4 + 7.5 μg LT3 daily for 90 days. Model-predicted plasma hormone responses to these treatments are clearly shown in **Figure 7**. The first 65 day prescription succeeded in normalizing TSH and bringing FT3 into the normal range, but FT4 overshot the normal range substantially. The second 85 day treatment normalized FT4 and TSH but failed to bring FT3 into the normal range. The combination therapy (last 90 days) succeeded in normalizing all three hormones.

## Discussion

The primary goal of this work was to update and refine the predictive abilities of original p-THYROSIM (1), a uniquely personalized simulation tool that mathematically mimics the thyroid hormone regulation system in humans (**Figure 1**). The original was developed to optimize replacement LT4 and LT4+LT3 dosing for hypothyroid patients, based on individual hormone levels, BMIs and gender. We followed the same original goals but use weight (*W*) and height (*H*) data independently – rather than BMI computed from *W* and *H*, in addition to sex, revised first as in **Figure 2**. We also made a few other key changes to the model that have rendered it more predictively accurate, eliminating an incorrect assumption about T3 degradation, and slightly tweaking the ranges of FT4 and FT3 to adjust for data and assay variability from different sources over different time periods used in model building.

Various effects of age, sex and anthropometric body size measurements on thyroid regulation or treatment have been reported in several publications in the recent past, e.g (9–11). But none except (1), to our knowledge, has formulated these measurements into a predictive model for simulating dynamic responses to anthropometric differences. The original p-THYROSIM (1) and our updated model described herein address this quantitative deficiency directly and are unique in this regard.

We have developed a very user-friendly iOS implementation of our updated model for the *iPhone* and *iPad*, rendering it maximally useful to the clinical community overall and to endocrinologists especially. As example clinical applications of the app, **Figures 4**-**6** illustrate the various pages of the app for user input and output hormone response results to: *hemi-thyroidectomy* (**Figure 4**); *optimal monotherapy vs. combination therapy* (**Figure 5**); and the ability to test up to *4 different dosing or testing strategies* and represent them simultaneously in a single comparative plot (3 superimposed in **Figure 6**). In **Figure 5**, it is apparent that 112.5 μg of LT4 in monotherapy (or even 125 μg LT4, not shown) do not alter FT3 levels very much, and are not as effective as adding LT3 in a combination dose of 100 μg LT4 + 10 (=2 x 5) μg LT3, which achieves mid-range for all hormones, consistent with regulated and safe use of LT3 in replacement therapy (12). The simulated female patient with more severe disease in **Figure 6** also shows combination dosing achieving better plasma hormone normalizations, while illustrating the ability of the app to superimpose multiple sets of responses.

**Figure 7** illustrates a second (Python) program version of the model, with time in days instead hours, to graphically represent the longer time-course of disease progression and treatment. This simulation illustrates hormone level predictions to potential treatments, in this case with both combination LT4+LT3 and LT4-only daily dosing, the former clearly more effective in normalizing all three hormone plasma levels. The Python code is also available at our Github: tinyurl.com/pthyrosim.

The new iOS version will be available from the Apple Store in the near future. All code is freely available at our Github (tinyurl.com/pthyrosim) for anyone to make their own application for the model over any time period. We are currently building another iOS app for the model in days (spanning1000 days) and an internet version of p-THYROSIM, for greater access to the app.

## Data Availability

The raw data supporting the conclusions of this article will be made available by the authors, without undue reservation.
